# DNA Content of Various Fluids and Tissues of the Human Body

**DOI:** 10.3390/genes15010017

**Published:** 2023-12-21

**Authors:** Jędrzej Siuta, Agnieszka Dobosz, Jerzy Kawecki, Tadeusz Dobosz

**Affiliations:** 1 Department of Forensic Medicine, Wroclaw Medical University, Mikulicza-Redeckiego 4, 50-345 Wroclaw, Polandtadeusz.dobosz@umw.edu.pl (T.D.); 2 Division of Basic Medical Science, Faculty of Pharmacy, Wroclaw Medical University, Borowska 211, 50-556 Wroclaw, Poland

**Keywords:** DNA content, DNA isolation, human tissues

## Abstract

Due to the scarcity of literature data on the DNA content of different human tissues, this study aimed to isolate DNA from different tissues and fluids of the human body together with the determination of its content in the samples studied. Material was collected and tests were performed between 1990 and 2010, during autopsies performed for prosecutor’s offices in the Department of Forensic Medicine. Goiter and thyroid cancer tissues were obtained from the Department of General Surgery, Gastroenterology and Endocrinology of Wroclaw Medical University. Isolated samples were measured spectrophotometrically, yielding an R 260/280 nm between 1.5 and 1.6. In some cases (when a sufficiently pure preparation could not be obtained), isolation was continued using the silica-based commercial QIAquick PCR Purification Kit (Qiagen). If the sampling tissues showed signs of decomposition such as bad odour or colour, the results were calibrated by Real-Time PCR, using the Quantifiler DNA assay (Thermo Fisher Scientific, Applied Biosystems). The results have shown that the maximum amount of genetic material was obtained from hair roots, adrenal glands, gonads and lymph nodes. The lowest DNA content per gram or milliliter of tissue or body fluid was found in adipose tissue, blood, saliva, bile, sweat, tears and the vitreous body of the eye. The presented findings indicate the best sources of high-quality DNA from the human body: gonads, kidneys, muscle (including heart), blood and bones (after decalcification).

## 1. Introduction

All scientific acts in molecular biology and medicine, ranging from microarrays, sequencing and genome writing to gene therapy, require high-quality deoxyribonucleic acid isolation. Although the same genetic code exists in every tissue of the human body, the practice shows that the utility of tissues for DNA preparation is different. It depends on the packing density of cell nuclei in the tissue. Other unfavorable factors include time since death, easy tissue access to bacteria, lack of repair enzyme activity, speed of autolytic processes, fermentation, etc. The decomposition of genetic material is exerted by endogenous enzymes which cause autolysis and the enzyme proteins of emerging micro-organisms which contribute to fermentation processes (resulting in acidification of the environment, and finally the defragmentation of a DNA molecule) [1,2].

The choice of both the appropriate DNA ‘source’ and the isolation method is of great importance in all departments of forensic medicine, where the genetic profile is often determined from samples found at a crime scene or taken at autopsy or exhumation. Depending on the type of biological material available, a specific method of human DNA quantification should be used.

Many methods are used to isolate and purify DNA, which differ not only in the procedure of execution but also in the quality of the results obtained [3,4,5]. According to the publication of Kinzinger and Holz [6], the salt method, taken for commonly tested muscles and blood, gives better results than the phenol–chloroform method. On the other hand, Caldarone and Buckley [7], in their study, concluded that the method for the quantification of DNA and RNA content using ethidium bromide as a fluorochrome is characterized by ease of execution, low detection threshold, possibility of automation and high sample throughput. Using the accelerated extraction method, the best-quality DNA was obtained from the brain, liver and lung. For postmortem DNA isolation, the stability of the genetic material depends on the time elapsed since death. According to Bär, Kratzer, Mächler and Schmid [8], the best-quality DNA (high yield and low fragmentation) can be obtained from the cerebral cortex, lymph nodes, and psoas major muscle up to 3 weeks after a death, while from the kidneys and spleen only up to 5 days after death. Very often, teeth and bones become the best source for DNA analysis after death because, compared to other tissues, they decompose most slowly [9]. Unfortunately, although DNA isolations from some tissues are performed almost routinely in departments of forensic medicine (among others), there is little data in the literature on the content of genetic material in each individual tissue and body fluid, and those that do appear are sometimes contradictory [10]. Although we can obtain DNA from all tested tissues and fluids of the human body, they differ in their content [2], as well as the rate of degradation of the genetic material; this depends on many factors, both external and internal. In this situation, we decided to finish our study and publish our data. The present study aimed to isolate DNA from various tissues and fluids of the human body with the determination of the amount of DNA that could be obtained from them and, if necessary, calibration of the results by real-time quantification method. The spectrophotometric method for the research was chosen, because it is well developed, widely used, very cheap and possible to be repeated in any laboratory. Our experience shows that spectrophotometric measurement of the quantity of DNA from the precisely prepared samples gives similar results compared to other methods (including qPCR). Additionally, data from the literature show that these differences do not exceed 5% [11,12]. Additional confirmation of some of the results using the qPCR method may be the basis for future research work.

The authors intend that this paper should not only be a report of the results of their research, but that it can also be a practical guide for other researchers. For this reason, the paper presents three examples of the practical use of the presented research results in real-life situations requiring the determination of DNA from human tissues in forensic practice. Additionally, the conclusions of this paper can provide practical guidance for researchers working on the described topics.

## 2. Materials and Methods

### 2.1. The Legal Basis

The legal basis for the research was ordered by the Public Prosecutor’s Office to carry out DNA identification tests from autopsied NN human cadavers.

### 2.2. Biological Material

Material was collected between 1990 and 2010 during autopsies performed at the Department of Forensic Medicine of Wroclaw Medical University. Goiter and thyroid cancer tissues were obtained during radical surgical interventions at the Department of General Surgery, Gastroenterology and Endocrinology in Wrocław. Due to the legal regulations in force in Poland at that time, goiter and thyroid cancer tissues were obtained as anonymous post-operative medical waste, initially intended for utilization. A total of 2117 blood samples, 500 bone samples, 157 skeletal muscle samples and between 4 and 50 samples of other tissues were tested.

### 2.3. DNA Isolation

All DNA isolations from post-mortem material were performed from tissues (from 0.5 g to 20 g of each) and fluids (from 0.3 mL to 25 mL) collected 48 to 72 h after death and used immediately after collection (in the same day) for DNA preparation. The exception was material obtained during autopsies carried out immediately before or during a day off from work (which may last from one to a maximum of three days in Poland)—in these cases material was frozen into −20 °C. The material was frozen immediately after its collection and was examined soon after its delivery the next working day. Samples of thyroid cancer and goiter were tissues collected intraoperatively from living people and examined right after surgery. Each extraction, for both tissue and fluid, was repeated at least four times. Quantification of samples was conducted contemporaneously with their collection. Fluids were diluted 1:1 with isotonic NaCl, vortexed, and centrifuged at 4000× *g*, for 5 min and then DNA was obtained from pellets. Tissues were cut into small pieces manually, and in cases where this was difficult, crushed in liquid nitrogen, either manually or in a cryogenic magnetic mill (SPEX 6860 CentriPrep Cryogenic Freezer/Mill, Thermo Fisher Scentific, Waltham, MA, USA). DNA was extracted using the classical Kunkel organic phenol technique [13] modified by the use of proteinase K instead of pronase. The remaining phenol (which may interfere with spectrophotometric quantification) was removed by double (repeated) precipitation by chloroform-isoamyl alcohol reagent. The final volume of collected DNA samples depended on the type of tissue and was 0.8–1.2 mL/1 g of tissue.

### 2.4. Quantification

Isolated DNA pellets after isoamyl alcohol precipitation were dissolved not in water, but in 0.05 M TE (TRIS/EDTA) buffer, which is suitable for both spectrophotometry and long-term freezing. Isolated samples were measured spectrophotometrically using the specialized spectrometer ‘GeneQuant’ (Pharmacia Biotech, Piscataway, NJ, USA), obtaining the proper R 260/280 nm between 1.5 and 1.6 (where R is absorbance measured at 260 nm divided by absorbance measured at 280 nm). Most of time normal (1 cm × 1 cm × 4 cm) quartz cuvettes were used, but if the DNA yield was small, we used thinner (0.5 cm × 1 cm × 4 cm or 0.2 cm × 1 cm × 4 cm) cuvettes. After checking for DNA purity and yield, practically all DNA solution was recovered from cuvettes to storage tubes, frozen, and awaited analysis. When the obtained preparation was not sufficiently pure (R 260/280 nm < 1.4 or >1.7), DNA isolation was repeated using a commercially available silica kit: QIAquick PCR Purification Kit (Qiagen, Hilden, Germany). If the cadaver showed signs of putrefaction, such as a purple or greenish colour or an unpleasant odor, collected samples were not subjected to spectrophotometric tests, but the concentrations of human DNA in a mixture of human, bacterial and fungal genetic material were determined using Real-Time PCR Quantifiler DNA assay (Thermo Fisher Scientific, Applied Biosystems, Waltham, MA, USA), strictly according to procedure instructions. The results were read on the ABI 7900 HT analyzer (Thermo Fisher Scientific, Applied Biosystems, Waltham, MA, USA). The level of DNA degradation was preliminarily checked using agarose gel electrophoresis with ethidium bromide.

The isolated genetic material was amplified using two identification kits based on STR marker analysis. The first identification kit (AmpFl SGM Plus PCR Amplification kit, Thermo Fisher Scientific, Applied Biosystems, Waltham, MA, USA) amplifies 10 STR loci plus the sex marker Amelogenin. The sizes of the amplicons included in this kit ranged from 98 bp to 360 bp. The second kit (AmpFl Identifiler Plus PCR Amplification Kit, Thermo Fisher Scientific, Applied Biosystems, Waltham, MA, USA) identified 15 STR loci plus the sex marker Amelogenin, and amplicon sizes also ranged from 98 bp to 360 bp.

## 3. Results

Generally, the fresh autopsy material was pure enough to obtain genuine results. According to the study by He et al., absorbance measurements with modern spectrophotometers are quick, no-cost and reliable for nucleic acid samples without contaminants [11]. The same conclusion was written by Nielsen et al. who confirmed that spectrophotometric results are very similar (±4%) to those obtained by, theoretically, more precise methods, such as real-time PCR [12].

The results obtained are summarized in Table 1, which presents the average values of DNA content in 1 g of tested tissue and 1 mL of fluid specimens. Relative differences between the results obtained from individual preparations from a given tissue or fluid never exceeded the upper limit of 15%. A similar table but in an incomplete version appeared in the student script shown in position [14] of the bibliography.

The results presented in Table 1 are generally in good agreement with other published results, when available [3,6,8,9,10]. A comparison of our results with other available results is presented in Table 2.

The tissue tested in the greatest quantities was blood, where 2117 samples were examined. The fewest studies (*n* = 4–10) were performed for cartilage, tendon, duodenum, cerebrospinal fluid, amniotic fluid and bile. The samples were tested spectrophotometrically and the absorbance A_260_/A_280_ ratio was used for quantification and purity checking calculations.

Nowadays, microvolume spectrophotometers are available that not only consume a very small amount of DNA ~1–2 µL, which represents a major advantage for samples that reveal much smaller DNA concentrations, but are also much less laborious and user-friendly, with automatic calculation of QC ratios.

Besides the presentation of the results of never tested tissues and body fluids (Table 1), the additional aim of this work was to draw attention to some additional problems that were observed during experiments. So far, only some of the most important tissues in forensic medicine, such as blood, bones and teeth, have been extensively studied. Other tissues were examined only sporadically, which increases the scientific value of the results presented in Table 1. The cerebrospinal fluid and amniotic fluid individual preparations gave such a wide range of results, that their results also had to be presented as ranges. Synovial fluid is a lubricant, a fluid present in the joints between moving bones, such as inside the knees, elbows or hips. It is hard to obtain, but is a decay-resistant source of relatively high-molecular-weight DNA. In human faeces, almost all obtained DNA came from the bacteria; human DNA has been detected in a very small amount only in the outer layer of the faeces, coming from the exfoliated intestinal epithelum.

It is worth noting that some tissues are difficult material to isolate due to the difficulty of homogenization—such as small intestine biopsy sections, prostate, skin, cord, placenta, adipose tissue or bile (which is surfactant-active and creates foam). Rib bones, which yield large amounts of DNA compared to many other samples tested, often suffer from serious degradation of the nucleic acid and should be decalcified as soon as possible to maximize yield. The statement of the fact of increased DNA degradation in ribs is one of the important original conclusions of this work. Ribs are extremely difficult and poorly repeatable material for proceeding, contrary to the petrous part of the temporal bone from the skull which is both DNA-rich and relatively resistant to degradation. For this reason, this bone is commonly considered as the best choice of human bone for DNA preparation. In some cases, the properties of the material and its composition contribute to the difficulties in obtaining good-quality DNA. Experiments performed by our research team have shown that some tissues homogenize more easily than others. The ability of the tissue to homogenize may be surprising, as some problems were expected for example with the skin, but not with the adipose tissue or placenta.

The autopsy material is quite fresh, and in most experiments, all amplicons were multiplied correctly, but occasionally some exceptions were observed. Amplification results obtained in several experiments ranged in product size from 103 bp (Amelogenin result) to 150 bp. Such fragment lengths indicate (with a high probability) severe DNA degradation. It is impossible to explain why such problems occur from time to time. Our advice for such troubles is simple: prepare and profile DNA extracted from different tissues from the same cadaver. For this reason, the rules of the autopsy lab recommend taking a core sample (for example, blood) for molecular purposes, as well as “reserve” tissue (for example, fragments of teeth or muscle).

Experiments have also shown that the liver contains endonucleases that degrade genetic material. The spleen, on the other hand, contains a lot of heme, which is an inhibitor of the PCR technique. The lung tissue material collected for the study had a lot of colloidal suspension in it, which negatively affected the spectrophotometric measurements and may be DNA adducts. Next, DNA extraction from some tumors is significantly more efficient with DTT. All formalin-fixed tissues are not recommended due to the problem of digestion by proteinase K—which has to be used in large quantities [14] and the occurrence of thymidine bridges between two DNA strands, which causes the great problems in PCR-based techniques. Unfortunately, formalin is still commonly used to preserve whole cadavers or individual organs. The consequences of this can be very serious. Formalin causes cancer, its strong odour causes coughing and lacrimation, contact with the skin (depending on the concentration) causes allergies or burns, and tissues in contact with it loses their natural colour after some time and take on an unpleasant grey-green ‘cadaveric’ hue. Ultimately, the seemingly most obvious and unrivaled use of formalin as a fixative for histological tissues was found to have the adverse effect of reducing the usefulness of paraffin blocks as a source of material for any possible (non-histological) study. For this reason, the replacement of formalin with glyoxal is suggested.

The results presented in Table 1 indicate that one of the best sources of DNA is the gonads. Our practice showed that DNA obtained from gonads presented a lack of degradation—in all cases, we obtained the big yield of excellent, non-degraded DNA, suitable to all used methods. It is worth mentioning that the content of genetic material in these organs is age-independent in humans. DNA was extracted and purified from the ovaries and testis from neonates as well as from elderly persons (>95 years of age). In the case of semen, isolation was performed without and with dithiothreitol (DTT). The results in Table 1 refer to the isolation without DTT which solubilized sperm heads. When DTT was applied, the amount of isolate increased almost two times [21]. When it comes to determining the degree of degradation of the obtained DNA it is explained that most of the DNA obtained in this research was used to determine forensic profiles. These were determined by multiplex assays covering a very wide range of amplicons. The analysis of the results of these tests allows for drawing very precise conclusions as to the degree of degradation of the tested DNA.

Finally, the following comment should be added to the data from Table 1: (1) It is not only the high DNA content that is important—for example, we discourage attempts to isolate DNA from hair roots (which have a high amount of DNA) because of the enormous labour involved. It was very hard to obtain a large enough sample—mostly, about 100 mg of hair roots was taken and the result was extrapolated. Additionally, the degree of hair DNA degradation strongly depends on the hair development cycle. (2) DNA received from the lungs is very dirty and cloudy and contains a colloidal suspension. (3) Due to its high enzyme content, the liver does not allow the production of high-molecular-weight DNA. (4) Due to the high level of heme residues (which are strong inhibitors in the PCR method) it is not recommended to prepare DNA from the spleen. (5) Blood samples must be collected on anticoagulant and mixed well. If a clot (even a very small one) occurs, the DNA yield dramatically decreases. (6) Adipose tissue, brain and marrow ‘blur’ during preparation. (7) Hard and fibrous tissues (e.g., bone, cartilage, intestine, prostate, tendons, fascia or skin) are very difficult to homogenise. (8) When taking DNA samples from tumors, you should bear potential problems in mind: during cancerogenesis, LOH (Loss of Heterozygosity, when we see only one allele) or MSI (Microsatellite Instability, when we see new alleles, created de novo) can be observed. Both LOS and MSI may make the identification of the paraffin block difficult or even impossible. (9) Formalin is still the most common agent used to preserve organs. It is not only very harmful to health (from causing coughing and tearing to carcinogenic effects) and causes loss of color of preserved tissues, but also reduces the usefulness of paraffin blocks as a source of material for most (except histological) research. Molecular tests showed that formalin physically fragments DNA (because it oxidizes to strong formic acid) and, worst of all, it may make PCR testing impossible because of preventing thermal melting of the DNA helix by binding both strands with strong thymidine bridges. For this reason, it is suggested to replace formalin with glyoxal.

At the end of this section, three examples of the practical use of the data from this article are presented. Very importantly, it is not only the use of DNA content given in Table 1, but also the use of the other data presented in the Results section of the article.

Example 1.

In 2001, the Department of Forensic Medicine in Wroclaw received a human placenta from the police, which had been found in a public dumpster. The placenta had been sealed in an airtight plastic container and immersed in an unknown liquid. Immediately upon opening the container, there was a strong smell typical of formaldehyde. After reading the comments about the destructive role of formalin, our lab technician decided to immediately remove the entire formalin solution and rinse the tissue. The liquid had to be removed as quickly as possible, so the placenta was immediately removed from the container, rinsed under running water, and then, to rinse the remaining formalin from the tissue, it was thrown into a container with plenty of water at pH 8.0. The excised fragment was treated like a bone, which means that it was homogenised in liquid nitrogen in a cryogenic bone mill. The resulting powder was digested with a buffer containing a digestion mixture, and proteinase K, DTT and DNA were isolated. This strategy proved to be fully effective and resulted in a complete DNA profile, including the heaviest amplicons. Other control samples taken from thinner parts of the placenta yielded a small amount of DNA as it was damaged by formalin.

Unfortunately, such a strategy is not always possible, as in another case, in which the victim’s human brain was stored by the perpetrator for many months in a jar of highly concentrated, highly acidic formalin; even after the tissue was washed out, it was not possible to establish a DNA profile even for the lightest amplicons.

Example 2.

In one of Wrocław’s student dormitories, an unknown perpetrator regularly left excrement in the staircase and other public areas of the building. Despite monitoring and increased checks, the perpetrator could not be detected for a long time. Eventually, a sample of the faeces was sent to the Department of Forensic Medicine in Wroclaw to attempt to establish the DNA of the perpetrator. After verifying the information described in this article—that faeces contain mainly bacterial DNA, with only small amounts of human DNA found mainly in its outer layer (which comes from the exfoliated epithelium of the intestine)—samples were taken from the outermost layer of the faeces. The determination of the genetic profile by PCR was successful. On the contrary, control samples taken from the inner parts of the faecal sample did not show enough human DNA for PCR. The story described ended with the dormitory manager publicly presenting the results of the faecal DNA test and announcing that, if the faeces reappeared, all residents would be subjected to mandatory DNA testing at the expense of the perpetrator thus detected. The method used proved successful, as the faeces never reappeared.

Example 3.

A headless neonatus, in very advanced decomposition, partially eaten by animals, was brought to the laboratory for DNA profiling. The problem was to select the most suitable tissue for the study. The data in Table 1 show that high-molecular-weight DNA is obtainable in similar cases from joint synovial fluid. Indeed, flushing out the joints allowed us to determine an almost complete DNA profile.

In all cases given above, taking into account the results in Table 1 (see examples), DNA profiles were obtained for forensic purposes. This proves that the given DNA preparation method is fully usable. On this basis, it can be said that the given DNA isolation method is fully refined and does not require further methodological changes and additions.

## 4. Discussion

Obtaining genetic material of satisfactory purity and quantity determines the success of further molecular biology procedures; therefore, the right choice of tissue or body fluid is an important part of many diagnostic procedures. Table 1, which provides a concise overview of the results of the study, includes different types of material, useful for both forensic and medical or biotechnological purposes. The materials tested were relatively fresh (48 to 72 h after death) and stored in a refrigerator; thus, the chances of obtaining a good-quality isolate were relatively high. For example, from gonads, there was a 100% success, which means that DNA was obtained from all tested gonads, regardless of the age of the person from whom the sample came. Moreover, the obtained DNA was not degraded and worked properly in all tests. It should be noted that for both live and post-mortem DNA sources, the time between obtaining the material and performing the isolation is a crucial element affecting the properties of the isolated product. Additionally, in the case of post-mortem material, the storage conditions of the cadaver are important. In light of the explorations made, it seems that the practical aspects of the pre-analytical phase may be a field of interest for many researchers.

The spectrophotometric measurement method was chosen for the following reasons: it was simple, quick, very cheap and sufficiently reliable [22]. Unfortunately, it is not possible to compare the results obtained with another advanced measurement method because, for reasons of economy, the qPCR test was performed only in a small number of cases in which the results of spectrophotometric measurements gave unreliable results.

Nowadays, DNA is the center of interest of almost all scientists, not only biologists and medical professionals. Despite these technical obstacles and problems, the problem of ‘DNA quality’ still occurs. There is no proper definition of ‘good DNA’: common opinion says that prepared DNA is ‘good’ when it has a high yield, small degradation and the ability to work in PCR techniques [23,24]. It is an excellent definition, but in our opinion, DNA is ’good’ when it is suitable (works properly) to all common methods used in molecular biology and medicine, such as CE (e.g., DNA profiling), digestion of DNA by various restriction enzymes, cloning obtained fragments into plasmids and others.

One of the aims of this work was the compilation of the general instructions for different researchers working on DNA. Based on experiments carried out at the Department of Molecular Techniques which is a part of the Department of Forensic Medicine in Wroclaw, depending on the purpose of the research to be performed, the following DNA sources should be chosen: 1. archaeology—bones (especially recommended is petrous part of temporal bone or teeth; 2. medicine: cadavers—gonads (ovaries or testes), living persons—blood or cheek swabs; 3. diagnostic procedures, e.g., molecular cause of diseases—fresh specimens surgical/intraoperative—any material can be taken.

Interestingly, only a few papers have been published so far on the effect of the described conditions on the quality of the isolate [6,7,10,13]. In turn, only Bär et al. [8] examined the post-mortem stability of DNA. Nowadays, one of the most widespread DNA isolation techniques is the silica method, although the phenol technique stubbornly refuses to recede into oblivion. The silica technique is based on the binding and elution of DNA previously bound on a column containing a silicon or glass filter. The affinity of the DNA to the bed is increased using chaotropic salts. Increasingly, kits containing magnetic beads to which suitable ligands are attached are also being used for automatic DNA isolation [25].

Based on the conducted research, conclusions can be drawn. Generally, the results presented in this paper are similar to those previously published in the literature [3,6,7,8,9,10,25,26] but our research team tested more samples and applied quite new material sources. Moreover, published results of other research teams present a too-small number of specimens to be ultimate.

Our findings have shown that the most robust source of DNA is hair roots, but it cannot be recommended, because depending on the growth stage of the hair, root material may have a broader range of DNA yield values than other tissues/fluids tested and results may thus be more variable per gram of tissue; also cutting the bulbs may be laborious, and telogenic hair is not useful. Blood, traditionally believed to be an excellent source of DNA, in the light of the research, is a poor source of DNA material; however, it is very stable and easy to obtain. The only nucleated blood cells are leukocytes and reticulocytes, and the efficiency of preparation is low. Additionally, if any clot (even very small) is present in the blood sample, the efficiency decreases significantly, because leucocytes can penetrate the clot and their DNA becomes unavailable for preparation. Human body cavity smears, especially from the rectum, not inserted in Table 1, have efficiency similar to skeletal muscles; however, they contain mostly bacterial DNA.

The results showed that the best compromise between easy preparation, high yield, purity and high molecular weight (lack of degradation) of DNA is gonads.

Finally, some additional remarks: First, the phenol/chloroform/isoamyl alcohol method used for DNA isolation is obsolete now. The better and cheaper approach is presented in papers [27,28]. Second, as our research has shown, the final results of DNA testing using the PCR method depend also on the type of polymerase used. The main conclusion of this paper is to highlight that to obtain the proper results for diagnostics, not only is the yield important, but also other factors, such as the degradation level of DNA. Additionally, the ‘usability’ of the DNA depends not only on the quantity but also on the possibility of performing diagnostics, such as, e.g., PRC and CE. For this reason, it is important to use all the information contained in the entirety of this article, not only the DNA quantity values presented in Table 1. The choice of the best tissue, or if there is no choice, information about which possible problems may occur, may be very useful and save time and money.

## Figures and Tables

**Table 1:** Average DNA content in different tissues and fluids of the human body.

**Solid Material (1 g)**	**Number of Samples (*n*)**	**DNA (μg) ± SD**
hair roots	19	4210 ± 37 *
adrenal gland	44	2425 ± 4 *
ovary	31	1900 ± 6 *
testis	44	1650 ± 13 *
lymph node	109	1190 ± 36 *
epididymis	65	925 ± 7 *
spongy bones, e.g., ribs	41	814 ± 10
skull (petrous part of temporal bone)	172	810 ± 27
compact bone, e.g., shaft of femoral bone	287	805 ± 15
cartilage	4	802 ± 5
tendon	4	800 ± 4
chorion	14	800 ± 17
cord	15	780 ± 12
liver	48	775 ± 23 *
placenta	17	750 ± 12
uterus	13	715 ± 10
small intestine	16	615 ± 15
spleen	20	500 ± 20 *
pancreas	17	455 ± 5 *
prostate	15	450 ± 4 *
duodenum	7	350 ± 5 *
thyroid cancer	22	270 ± 12
heart	68	265 ± 4 *
skin	60	260 ± 5 *
salivary gland	75	260 ± 9 *
thyroid cancer, FFPE	37	250 ± 9
ovary cancer (HeLa cells)	9	250 ± 3
thyroid gland	13	245 ± 14 *
thyroid goiter	9	240 ± 10
kidneys	19	225 ± 9*
lungs	16	225 ± 6
single whole human tooth	20	220–225 (depends on the kind of tooth; more in molar teeth)
dental pulp (totally decalcified, average in 1 g of pulp)	10	200 ± 1
skeletal muscles	157	170 ± 7 *
brain	78	110 ± 14
adipose tissue	78	80 ± 6 *
faeces **	10	45 ± 1 (mainly *Escherichia coli* DNA) **
iris	5	1
retina	5	5
vitreous body of the eye	5	1 *
**Liquid Material (1 mL)**	**Number of Samples (*n*)**	**DNA (μg)**
semen with normospermia	52	1300 ± 12 with DTT [15]
semen with normospermia	42	660 ± 14 *
synovial fluid	16	100 ± 4
blood (with anticoagulant)	2117	33 ± 7 *
centrifuged cerebrospinal fluid	10	2–5 ($\bar{x}=$ 3.30)
centrifuged amniotic fluid	10	1–4 ($\bar{x}=$ 2)
bile	5	3 *
centrifuged saliva	10	1
lymph	10	1
sweat and centrifuged urine	10	<1
tears	10	<<1
gastric juice	10	0 (extremely degraded DNA)

* Preliminary data were published for student use in Polish (2014) [14]. ** *E. coli* DNA was determined by its own RT-PCR method. Technical details can be sent on demand by the corresponding author.

**Table 2:** Comparison of our results with other published results.

Tissue	Our Result	Results of Other Authors
muscle	170	230 [3,8,15] *
brain	20	20–40 [6,16] *
dura	2.5	2.5 [17]*
placenta	750	340 [18] *
blood	33	30–60 [6,8,9,10] *
buccal swab	max 250/swab	54 [19] *
iris	1	0.2 [20] *
retina	5	3 [20] *

* Number of the work cited in references.

## Data Availability

Data presented in this study are partially available in Polish script for students [Dobosz, T. Chapter 5: Genetyka sądowa; Izolacja DNA i RNA. In Wybrane Zagadnienia z Medycyny Sądowej; Żaba, C., Ed.; Wydawnictwo Naukowe Uniwersytetu Medycznego im. Karola Marcinkowskiego w Poznaniu: Poznań, Poland, 2014; p. 76.]. The rest of the data can be collected from Authors upon request.
